# Stability of wheat grain yields over three field seasons in the UK

**DOI:** 10.1002/fes3.147

**Published:** 2018-09-19

**Authors:** João Paulo Pennacchi, Elizabete Carmo‐Silva, P. John Andralojc, Tracy Lawson, Alexandra M. Allen, Christine A. Raines, Martin A. J. Parry

**Affiliations:** ^1^ Lancaster Environment Centre Lancaster University Lancaster UK; ^2^ Plant Biology and Crop Science Rothamsted Research Harpenden UK; ^3^ Department of Biological Sciences University of Essex Colchester UK; ^4^ School of Biological Sciences University of Bristol Bristol UK

**Keywords:** breeding, climate change, crop productivity, food security, genetic variation, grain yield, heritability, suboptimal conditions, *Triticum aestivum*, yield stability

## Abstract

Ensuring food security in a changing climate is a major contemporary challenge and requires development of climate‐resilient crops that perform well under variable environments. The hypothesis that yield stability in suboptimal conditions is linked to yield penalties in optimal conditions was investigated in field‐grown wheat in the UK. The phenotypic responses, rate of wheat crop development, and final grain yield to varying sowing date, rainfall, air temperature, and radiation patterns were studied for a panel of 61 elite commercial wheat cultivars grown in the UK in 2012, 2013, and 2014. Contrasting climatic patterns, particularly rainfall accumulation and distribution over the season, influenced the relative performance of the cultivars affecting the duration of grain development stage and impacting on productivity. Indices for crop productivity, yield stability, and performance under suboptimal conditions revealed four cultivars with a combination of stable and high relative grain yields over the three seasons: Gladiator, Humber, Mercato, and Zebedee. Genetic similarity between cultivars partially explained yield performance in the contrasting seasons. The year of release of the cultivars correlated with grain yield but not with yield stability, supporting the contention that breeding for yield potential does not select for climate resilience and yield stability of crops. Further analysis of the outstanding cultivars may unravel target traits for breeding efforts aimed at increasing wheat yield potential and stability in the changing climate.

## 1 INTRODUCTION

Arable crop research plays an important role in the context of sustainable and environmentally friendly food production. Advances in crop science have contributed greatly to improve food security by keeping food availability ahead of demand (Gregory & George, 2011). However, ensuring food security in the near future is challenging, mainly considering the predicted scenarios of growing world population (Godfray et al., 2010), changes in food consumption patterns (Pingali, 2006), extreme climatic events (Tilman & Clark, 2015), and the need for sustainable use of resources in agricultural activities (Berry, Dernini, Burlingame, Meybeck, & Conforti, 2015).

Climatic volatility greatly influences crop development and yields (Schmidhuber & Tubiello, 2007) with climatic factors accounting for one‐third of crop yield variability (Ray, Gerber, MacDonald, & West, 2015). In wheat, high temperatures combined with limited water supply at critical growth stages are recognized to be a major cause of yield loss (Ciais et al., 2005). Improving wheat yield stability and ensuring crop performance under suboptimal conditions are crucial for food security as the crop represents 20% of the caloric intake of the world's population (Braun, Atlin, & Payne, 2010). The Green Revolution has been successful in increasing wheat yield potential, that is, the yield of a cultivar grown under optimal environmental conditions, with ideal availability of nutrients and water, and control of biotic and abiotic stresses (Evans & Fischer, 1999). At the farm scale, the ideal growth conditions for achieving yield potential are rarely observed, although there are multiple management technologies which can minimize predictable climatic impacts (Robertson, Kirkegaard, Rebetzke, Llewellyn, & Wark, 2016). The difference between the yield potential and the on‐farm yield is known as the yield gap (Fischer, Byerlee, & Edmeades, 2014; Lobell, Cassman, & Field, 2009). Modern climatic challenges to crop production mean that current and future efforts in crop breeding must continue to increase yield potential while decreasing the yield gap, that is, ensuring that farm yields are commensurate with the yield potential, despite the observed climatic conditions (Araus, Slafer, Royo, & Serret, 2008).

Understanding yield stability and crop performance under suboptimal conditions is key to decreasing the yield gap (Fischer & Edmeades, 2010). Stability is defined as the ability of a given genotype to perform consistently across different environments and years of cultivation (Romagosa & Fox, 1993). Crop performance under suboptimal conditions can be related to multiple biotic and abiotic factors. In the scope of this study, crop performance was evaluated under contrasting conditions of rainfall accumulation and distribution, and evapotranspiration demand, as a combined effect of air temperature and humidity. In general, the UK is representative of well‐watered winter wheat cultivation (Fischer & Edmeades, 2010). For the purpose of the present study, yield stability is considered a general characteristic of a genotype over multiple seasons or environments.

Some studies suggest that crop performance under suboptimal conditions is linked with yield penalties in years of optimal conditions (Tester & Langridge, 2010). Identifying cultivars with combined high and stable yields and characterizing the genetic and physiological background of yield potential, stability, and performance under suboptimal conditions could enhance the understanding of the different strategies to reach improved yield performance despite the climatic conditions observed in any given season (Reynolds & Langridge, 2016).

The present study aimed to test the previously suggested hypothesis that yield stability in suboptimal conditions is linked to yield penalties in optimal conditions (Tester & Langridge, 2010), in a panel of commercial wheat cultivars in the UK. An additional objective was to investigate the impact of breeding over the last decades on grain yield and stability. The impact of variable environmental conditions over three consecutive field seasons on crop development and grain yield was evaluated. The results support the contention that contrasting climatic patterns, particularly rainfall accumulation and distribution over the growing season, influenced the crop development rate and relative grain yield patterns. Moreover, the combined results suggest that grain yield and crop performance under variable environments are not mutually exclusive traits. The results also suggest that breeding has favored yield potential without a concomitant improvement of yield stability. Four of the 61 cultivars delivered combined high and stable yields over the three seasons. Further investigation of traits presented in these cultivars can inform the breeding of high yielding and climate‐resilient wheat cultivars to ensure future food security in the changing climate.

## 2 MATERIALS AND METHODS

### 2.1 Plant material and field experiments

The ERYCC panel is composed of 64 wheat elite cultivars, mainly from France and the UK, released between 1975 and 2008, and selected for Earliness and Resilience for Yield in a Changing Climate (ERYCC) (Ober et al., 2013). The panel assembly was part of a project involving the Agriculture and Horticulture Development Board (AHDB) and funded by a DEFRA (Department for Environment, Food & Rural Affairs) Sustainable Arable Link to characterize wheat cultivars for earliness and resilience traits and identify potential parents for further crosses (Clarke et al., 2012).

Plants of 61 ERYCC wheat cultivars were grown at the Rothamsted Research farm, in Harpenden, UK, for three consecutive seasons, the first being harvested in 2012 and the last in 2014. Seeds for the first experiment were acquired from UK breeders, and, for the following experiments, the seed used was that harvested from the previous experiment. All the experiments were planted as first wheat crops with sowing rate of 350 seeds per m^2^, in three randomized blocks. Detailed information specific to each experiment is presented below (experiments identified by year of harvest):

- 2012: experiment planted at the Great Field 1&2, in a typical Batcombe soil (Avery & Catt, 1995) after oilseed rape crop, in 2 × 1 m plots (2 m^2^), sown on 05/10/2011 and harvested on 17/08/2012 (Driever, Lawson, Andralojc, Raines, & Parry, 2014).
- 2013: experiment planted at the Black Horse Field, in a Charity–Humble soil (Avery & Catt, 1995) after oat crop, in 3 × 1 m plots (3 m^2^), sown on 12/12/2012 and harvested on 28/08/2013 (Carmo‐Silva et al., 2017).
- 2014: experiment planted at the Little Hoos Field, in a typical Batcombe soil (Avery & Catt, 1995), after oilseed rape crop, in 9 × 1.8 m plots (16.2 m^2^), sown on 15/11/2013 and harvested on 22/08/2014.

In 2012 and 2013, the 64 ERYCC wheat cultivars were grown. In 2014, three cultivars of the ERYCC panel (Cappelle Desprez, Deben, and Mercia) were replaced by other two more modern wheat cultivars and a triticale cultivar. The replacement aimed to compare the performance of recently released cultivars to the rest of the panel in the 2014 season (data not shown). Data analysis herein considered the 61 cultivars that were grown over the three seasons. Information about date of cultivar release, origin, habit, market type, grouping, and parentage of the 61 studied cultivars is presented in Supporting Information Table S1.

Application of fungicides, insecticides, and herbicides, as well as fertilizers, was done accordingly to Rothamsted farm practices in the three seasons (Supporting Information Table S2).

### 2.2 Meteorological data

The meteorological data were acquired from the Rothamsted Meteorological Station at the Rothamsted farm. The distance from the station to the experiments was, in a straight line, of: 100 m for the 2012 experiment, 2.5 km for the 2013 experiment, and 1.3 km for the 2014 experiment. The maximum and minimum daily temperature (°C), the daily rainfall (mm), and the radiation (MJ/m^2^) were used. From these data, the accumulated rainfall and accumulated radiation for a specific period were calculated as the sum of the daily value from the first to the last day in the period considered. Average daily temperature (*T*_med_) was calculated as the mean of maximum daily temperature and minimum daily temperature. Degrees day was calculated considering the base temperature (*T*_base_) for wheat crop as zero (McMaster & Smika, 1988) and by the equation system below:

(1) $$\text{Degrees}\;\text{day}=\left\{\begin{array}{cc} T_{\mathrm{med}}-T_{\mathrm{base}}, & \text{if}\;T_{\mathrm{med}}>T_{\mathrm{base}}\\ 0, & \text{if}\;T_{\mathrm{med}}\leq T_{\mathrm{base}} \end{array}\right..$$

The accumulated degrees day for a period of time was calculated as the sum of the degrees day from the first to the last day in the considered period.

### 2.3 Crop development monitoring and growth stage definition

The Zadoks scale (Zadoks, Chang, & Konzak, 1974) was used to assess the date when half of the plants in each plot attained a given cereal growth stage. The scale is based on scores relative to crop development stages: tillering, stem elongation, booting, flag leaf expansion, ear emergence, flowering, grain filling, and maturation. The frequency of crop development monitoring depended on the crop stage and rate of change, being more frequent when crop development was faster and less frequent when crop development was slower. The delay in sowing was calculated as the number of days between sowing and the limit date for early sowing of winter wheat in UK, September 15 (AHDB, 2011).

### 2.4 Yield measurement

Plants were harvested using a Haldrup‐C65 (Haldrup, Le Mans, France) plot combine. Grain and straw weights were measured by the combine and corrected to 100% dry matter based on moisture content of a subsample taken from the harvested plot, at harvest time. Harvest index was calculated by the ratio of grain to total aboveground biomass weight (grain + straw) at 100% dry matter. Linear mixed models were fitted to the data corresponding to each year independently to evaluate any effects of possible spatial heterogeneity in crop yield. This analysis evaluated possible effects of rows and columns of the experimental field on the covariance structure of grain yield residuals (Cullis, Smith, & Coombes, 2006).

Relative values of grain yield, biomass, and harvest index were calculated for each cultivar by dividing the measured value for the cultivar by the average value of the 61 cultivars in the respective year. By way of example, a relative grain yield value of 1 means that the cultivar had the same grain yield as the average for the 61 cultivars in that season.

### 2.5 Productivity, stability, and performance under suboptimal conditions

The following indices were calculated for each cultivar: (a) the productivity index was calculated as the average of the relative grain yield over the three seasons; (b) the yield stability index was calculated as the ratio between the cultivar grain yield standard deviation and the average grain yield for the 61 cultivars over the three seasons; (c) the suboptimal performance index was calculated as the average of the relative grain yield for 2013 and 2014, due to the lower accumulated rainfall at crucial stages and the reduction in grain development duration. Index values were ranked from 1 to 10, with the smallest value being ranked 1 and the highest ranked 10; the intermediate values were calculated based on a linear regression between the minimum and maximum limits defined by a first‐degree equation.

### 2.6 Year of release analysis

Correlation analysis and linear regression were used to evaluate the impact of the year of release on cultivar productivity and stability. Four different compositions of the population were analyzed according to year of release: ERYCC panel (full population—61 cultivars), post‐1980 (cultivars released after 1980—56 cultivars), post‐1990 (cultivars released after 1990—45 cultivars) and post‐2000 (cultivars released after 2000—36 cultivars). These compositions aimed to study the impact of breeding on productivity and yield stability.

### 2.7 Genotyping and genetic similarities

Grain subsamples for the 61 cultivars were taken from the 2012 harvest for genotyping. The Axiom^®^ Wheat Breeder's Array was used at the School of Biological Sciences, University of Bristol, to genotype the cultivars using the Affymetrix GeneTitan^®^ system, according to the procedure described by Affymetrix (Axiom^®^ 2.0 Assay Manual Workflow User Guide Rev3). A total of 35,143 markers were screened for the 61 cultivars. Allele calling was carried out using the Affymetrix proprietary software package Affymetrix Analysis Suite. The genetic distance (GenDist) for pairs of cultivars was calculated according to Gao, Yang, Zhao, and Pan (2005). From the genetic distance matrix, a similarity matrix was calculated by:

(2) $${\text{Similarity}}_{\mathrm{ab}}=1-{\text{GenDist}}_{\mathrm{ab}},$$

where *a* and *b* are the two cultivars for which the similarity is being measured.

A hierarchical cluster analysis based on group average was carried out for the similarity matrix, using GenStat 17th Edition (VSN International Ltd., Hemel Hempstead, UK). The similarity to Gladiator was used for the correlation analysis, due to its superior performance in terms of the average relative grain yield over the three seasons.

### 2.8 Heritability

Broad‐sense heritability (H^2^) was calculated for grain yield in each season using the procedure described by Cullis et al. (2006), based on the ratio of the between‐cultivar variance component and the mean variance of the difference between two cultivar means, as estimated by best linear unbiased predictors (BLUPs).

### 2.9 Correlation analysis

The Pearson product–moment (PPM) coefficients (*r*) were used to assess correlations between traits using GenStat 17th Edition (VSN International Ltd.).

## 3 RESULTS

### 3.1 The duration of the crop development phases was affected by sowing date and climatic conditions

**Figure 1:**
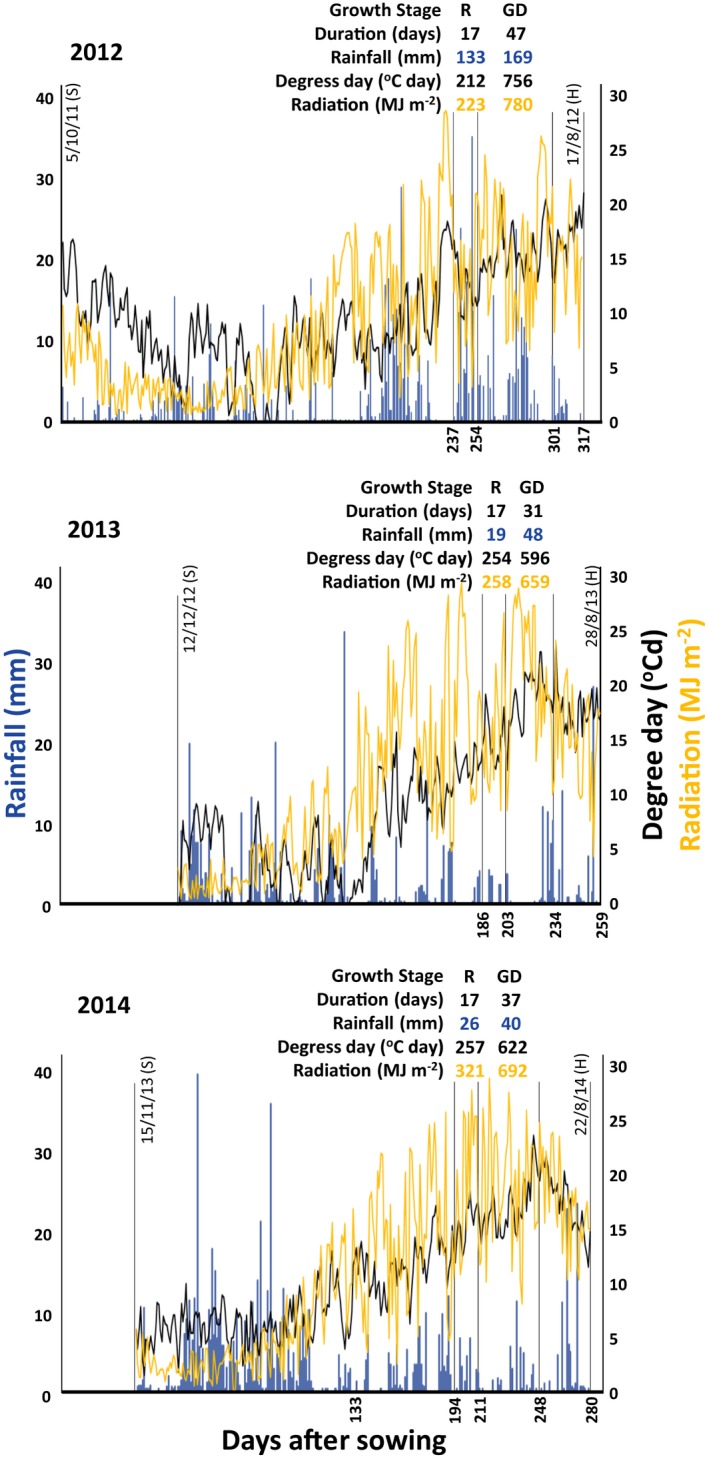
Meteorological and developmental data for 61 UK field‐grown wheat cultivars over three consecutive seasons (2012, 2013, and 2014). The dates at the left‐ and right‐hand side of the graphs correspond to each season's sowing (S) and harvest (H) dates, respectively. The left *y*‐axis in each graph starts at the day of the earliest sowing (October 5), and the right *y*‐axis finishes at the day of the latest harvest (28th of August). Growth stages defined according to Zadoks scale (Zadoks et al., 1974): R, reproductive (Z5.0 to Z7.0); GD, grain development (Z7.0 to Z9.0)

The crop growing season was longer in 2012 (317 days) than in 2014 (280 days) and was shortest in 2013 (259 days). In all three growing seasons, for logistic reasons, the crop was sown later than recommend for winter wheat crops in the UK (15th September; AHDB, 2011). Sowing in the 2013 season was 88 days late (12/12/2012; Figure 1), followed by the 2014 season, which was 61 days late (15/11/2013), and the 2012 season, which was 20 days late (5/10/2011). The grain development stage was particularly shorter, with 31 days in 2013, compared to 37 days in 2014 and 47 days in 2012 (Figure 1). In an integrated analysis of data over the three seasons, the delay in sowing was negatively correlated to the duration of the grain development phase (*r *=* *−0.93, *p* < 0.001).

The three seasons were characterized by considerably different meteorological conditions, which may have impacted on the duration of the crop development phases (Figure 1). The 2012 season was characterized by the highest rainfall accumulation, over the whole season (768 mm) and during the vegetative growth (201 mm), reproductive (133 mm), and grain development stages (169 mm). The 2013 season had the lowest rainfall accumulated over the whole season (506 mm) and during the vegetative growth (75 mm), reproductive (19 mm), and grain development stages (48 mm). For the 2014 season, the total rainfall accumulated was closer to the 2012 season (739 mm), but unevenly distributed over the growing season, with greater accumulation at the early stages. The rainfall accumulated in 2014 at the vegetative growth (115 mm), reproductive (26 mm), and grain development stages (40 mm) was much lower than in 2012 and closer to the 2013 patterns (Figure 1). The final radiation accumulated was very similar for the three seasons; however, 2014 presented higher accumulated radiation at the reproductive stage. On the contrary, the accumulated degrees day over the season was much lower in 2013 (2,411°C day) than in 2012 and 2014 (3,114 and 2,948°C day, respectively). Overall, both accumulated rainfall and degrees day were fairly well distributed over the 2012 season, while in 2013 and 2014, rainfall was less frequent and degrees day and radiation accumulated to a greater extent at the later developmental stages. The greater accumulated rainfall during the establishment and vegetative growth stages could have provided sufficient soil moisture for sustained development of the crop in 2014 compared to 2013.

### 3.2 Breeding of modern wheat cultivars improved productivity, but not stability

The average grain yield of the 61 cultivars was highest in 2012 (12.3 t/ha), intermediate in 2014 (11.6 t/ha), and lowest in 2013 (7.9 t/ha). The relative grain yield of each cultivar, as a fraction of the average yield of the 61 cultivars in each season (Table 1), provides an assessment of the impact of the different sowing dates and environmental conditions experienced in each season over cultivar‐specific plant performance. The relative yield performance pattern was more similar between 2013 and 2014, than between either of these years and 2012. Accordingly, the relative grain yields in 2013 and 2014 were more strongly correlated (*r *=* *0.44, *p* < 0.01), than the grain yields in 2012 and 2013 (*r *=* *0.25, *p* = 0.06) or 2012 and 2014 (*r *=* *0.26, *p* < 0.05). The similarity in relative grain yield patterns for 2013 and 2014 could have resulted from the later sowing date and less regular distribution of rainfall in later developmental stages in these two seasons compared to 2012 (Figure 1). The suboptimal conditions were more pronounced in 2013, resulting in greater variability in grain yields and lower broad‐sense heritability compared to 2012 and 2014 (Table 2), and suggesting greater genetic control of grain yields in 2012 and 2014 compared to 2013.

**Table 1:** Grain yield of 61 UK wheat cultivars relative to the panel average for each of three seasons (2012, 2013, and 2014). Values are means ± standard error of the mean (*n* = 3) of plot grain yield for a given cultivar as a ratio of the respective season average grain yield for the 61 cultivars. Green arrows directed upward represent values on the upper quartile; yellow arrows directed to the right represent values on the two intermediate quartiles; red arrows directed downward represent values on the lower quartile. Cultivars in light gray had relative grain yields always at or above the 61‐cultivar average; cultivars in dark gray had relative grain yields always at or above the 61‐cultivar average and were in the upper quartile for 2013 and 2014

Cultivar	Relative Grain Yield + *SEM*					
	2012		2013		2014	
Access	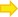	1.02 ± 0.04	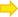	1.06 ± 0.11	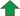	1.05 ± 0.02
Alchemy	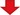	0.91 ± 0.04	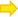	0.94 ± 0.11	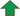	1.06 ± 0.02
Alixan	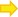	0.98 ± 0.04	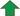	1.08 ± 0.11	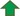	1.07 ± 0.02
Ambrosia	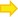	1.02 ± 0.04	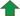	1.13 ± 0.11	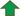	1.08 ± 0.02
Andalou	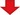	0.78 ± 0.04	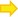	0.99 ± 0.11	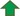	1.05 ± 0.02
Apache	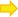	1.02 ± 0.04	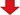	0.81 ± 0.11	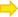	1.00 ± 0.02
Avalon	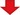	0.90 ± 0.05	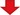	0.75 ± 0.11	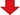	0.93 ± 0.02
Bacanora	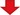	0.80 ± 0.04	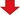	0.87 ± 0.11	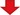	0.91 ± 0.02
Battalion	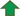	1.12 ± 0.04	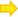	1.04 ± 0.11	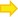	0.98 ± 0.02
Beaver	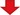	0.90 ± 0.04	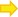	1.06 ± 0.11	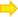	1.02 ± 0.02
Brompton	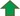	1.10 ± 0.04	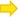	1.00 ± 0.11	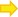	1.02 ± 0.02
Buster	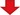	0.84 ± 0.04	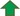	1.22 ± 0.11	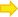	0.99 ± 0.02
Cadenza	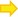	1.01 ± 0.04	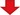	0.77 ± 0.11	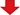	0.95 ± 0.02
Caphorn	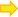	0.99 ± 0.04	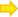	1.05 ± 0.11	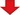	0.94 ± 0.02
Cezanne	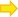	1.03 ± 0.05	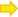	1.04 ± 0.11	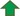	1.05 ± 0.02
Claire	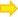	1.02 ± 0.04	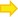	0.91 ± 0.11	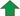	1.06 ± 0.02
Consort	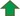	1.17 ± 0.04	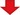	0.89 ± 0.11	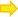	0.95 ± 0.02
Cordiale	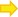	1.07 ± 0.04	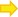	0.9 ± 0.11	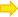	1.03 ± 0.02
Dover	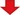	0.93 ± 0.04	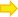	0.95 ± 0.11	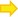	0.98 ± 0.02
Einstein	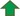	1.09 ± 0.04	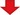	0.88 ± 0.11	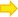	1.01 ± 0.02
Equinox	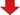	0.79 ± 0.04	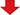	0.84 ± 0.11	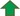	1.06 ± 0.02
Exotic	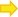	0.96 ± 0.04	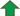	1.09 ± 0.11	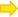	1 00 ± 0.02
Exsept	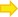	1.06 ± 0.04	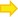	0.99 ± 0.11	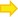	0.98 ± 0.02
Galahad	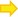	1.03 ± 0.04	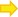	1.00 ± 0.11	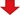	0.94 ± 0.02
Gatsby	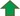	1.19 ± 0.04	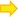	0.95 ± 0.11	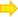	0.98 ± 0.02
Gladiator	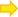	1.05 ± 0.04	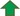	1.29 ± 0.11	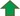	1.13 ± 0.02
Glasgow	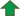	1.09 ± 0.04	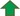	1.21 ± 0.11	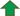	1.07 ± 0.02
Gulliver	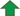	1.10 ± 0.04	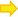	1.06 ± 0.11	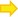	0.99 ± 0.02
Haven	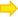	0.97 ± 0.04	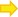	1.05 ± 0.11	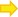	1.03 ± 0.02
Hereward	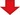	0.85 ± 0.05	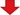	0.85 ± 0.11	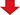	0.92 ± 0.02
Hobbit	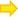	0.97 ± 0.05	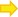	1.02 ± 0.11	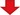	0.95 ± 0.02
Humber	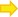	1.04 ± 0.04	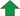	1.22 ± 0.11	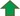	1.11 ± 0.02
Huntsman	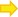	1.05 ± 0.04	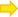	1.01 ± 0.11	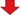	0.93 ± 0.02
Hustler	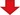	0.82 ± 0.04	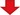	0.82 ± 0.11	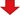	0.94 ± 0.02
Hyperion	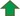	1.11 ± 0.04	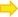	1.02 ± 0.11	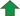	1.07 ± 0.02
Istabraq	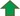	1.19 ± 0.04	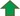	1.10 ± 0.11	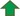	1.10 ± 0.02
Longbow	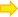	0.98 ± 0.04	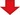	0.85 ± 0.11	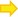	1.03 ± 0.02
Malacca	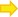	0.94 ± 0.05	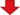	0.89 ± 0.11	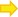	0.99 ± 0.02
Maris Widgeon	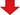	0.84 ± 0.04	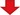	0.87 ± 0.11	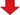	0.66 ± 0.02
Marksman	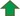	1.09 ± 0.04	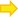	0.94 ± 0.11	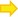	1.01 ± 0.02
Mascot	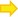	1.02 ± 0.04	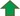	1.11 ± 0.11	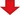	0.95 ± 0.02
Mendel	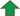	1.14 ± 0.04	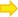	1.02 ± 0.11	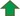	1.07 ± 0.02
Mercato	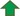	1.10 ± 0.04	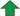	1.30 ± 0.11	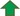	1.04 ± 0.02
Musketeer	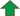	1.11 ± 0.04	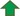	1.09 ± 0.11	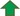	1.05 ± 0.02
Norman	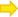	0.96 ± 0.04	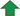	1.09 ± 0.11	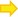	1.01 ± 0.02
Oakley	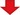	0.94 ± 0.04	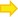	1.05 ± 0.11	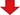	0.94 ± 0.02
Paragon	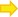	1.00 ± 0.04	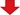	0.81 ± 0.11	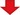	0.95 ± 0.02
Recital	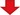	0.62 ± 0.04	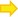	1.05 ± 0.11	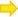	1.03 ± 0.02
Rialto	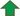	1.15 ± 0.04	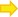	0.99 ± 0.11	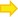	0.98 ± 0.02
Riband	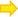	0.98 ± 0.04	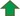	1.09 ± 0.11	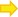	0.99 ± 0.02
Robigus	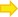	0.99 ± 0.04	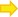	0.94 ± 0.11	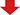	0.91 ± 0.02
Royssac	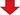	0.87 ± 0.04	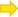	0.91 ± 0.11	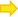	1.00 ± 0.02
Sankara	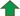	1.09 ± 0.04	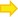	1.07 ± 0.11	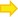	1.04 ± 0.02
Savannah	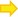	1.07 ± 0.04	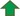	1.10 ± 0.11	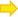	1.02 ± 0.02
Soissons	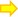	1.01 ± 0.04	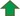	1.16 ± 0.11	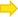	1.00 ± 0.02
Solstice	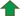	1.10 ± 0.04	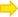	0.94 ± 0.11	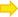	0.98 ± 0.02
Spark	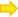	1.02 ± 0.04	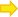	0.89 ± 0.11	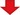	0.95 ± 0.02
Timber	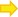	1.02 ± 0.05	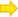	0.98 ± 0.11	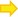	1.04 ± 0.02
Virtue	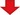	0.84 ± 0.04	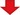	0.8 ± 0.11	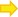	0.99 ± 0.02
Xi19	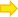	1.06 ± 0.04	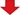	0.89 ± 0.11	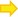	1.02 ± 0.02
Zebedee	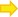	1.00 ± 0.04	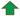	1.30 ± 0.11	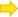	1.04 ± 0.02

**Table 2:** Standard error of the mean (*SEM*) and heritability for grain yield of 61 UK field‐grown wheat cultivars over three seasons (2012, 2013, and 2014)

	2012	2013	2014
*SEM*	0.04	0.11	0.02
Heritability	0.86	0.74	0.82

**Figure 2:**
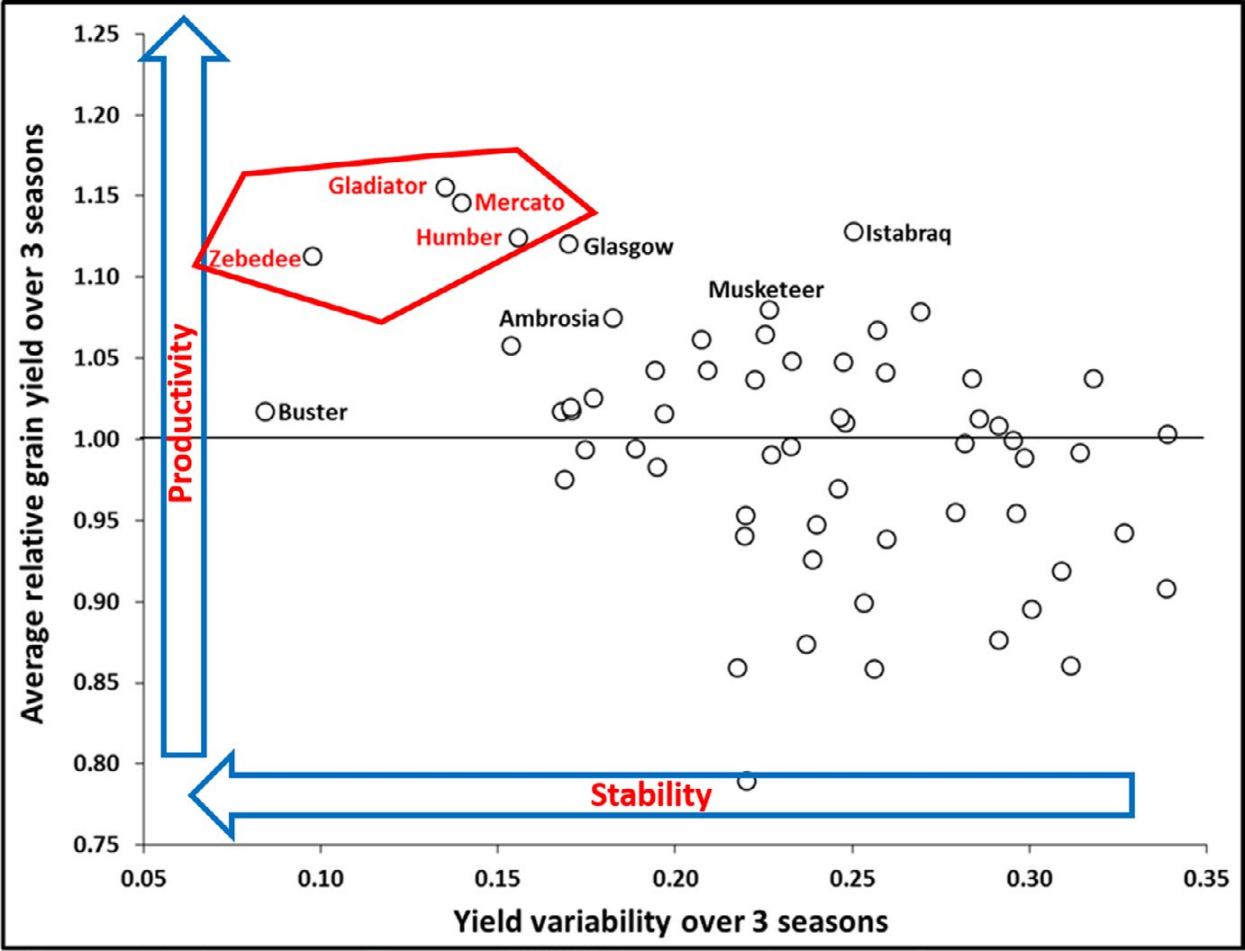
Relationship between grain yield and yield stability of 61 UK field‐grown wheat cultivars over three consecutive seasons (2012, 2013, and 2014). The horizontal line represents the average for the 61 cultivars over the three seasons

Of the 61 cultivars, 16 always yielded at or above the average (highlighted in gray; Table 1). From those, Gladiator, Humber, Mercato, and Zebedee presented high and stable yields over the three years (Figure 2). These cultivars also presented an improved performance under suboptimal conditions (2013 and 2014), with Zebedee close to the higher quartile for 2014 (1.04 relative yield compared to 1.05 as the top quartile baseline; Table 1). There was a positive correlation between productivity and stability for the panel over the three seasons (*r *=* *0.40, *p* < 0.001; Figure 2).

**Figure 3:**
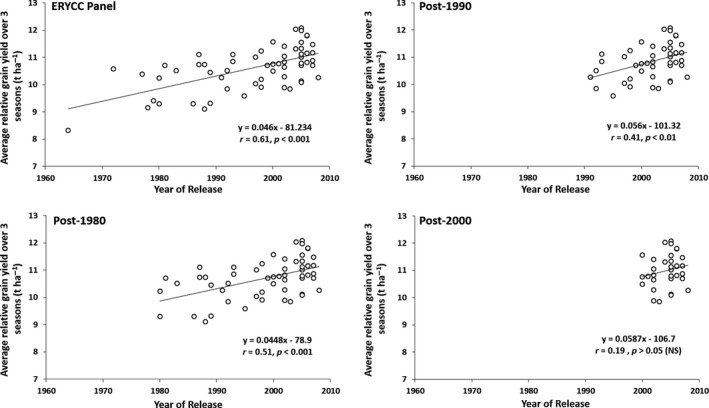
Linear regression between year of release and average relative grain yield for different conformations of the ERYCC panel wheat population grown in the UK over three consecutive seasons (2012, 2013, and 2014). ERYCC panel, full population (*n* = 61 cultivars); post‐1980, cultivars released after 1980 (*n* = 56 cultivars); post‐1990, cultivars released after 1990 (*n* = 45 cultivars); post‐2000, cultivars released after 2000 (*n* = 36 cultivars). Pearson's correlation coefficients (*r*) at a *p* significance given by a *F* test

The oldest cultivar of the ERYCC panel was released in 1964 (Maris Widgeon) and the newest in 2008 (Oakley), with other cultivars having been released over the decades in between. The year of release was positively correlated to grain yield in each year (2012, *r *=* *0.47, *p* < 0.001; 2013, *r *=* *0.33, *p* < 0.01; 2014, *r *=* *0.56, *p* < 0.001) and over the three seasons (*r *=* *0.61, *p* < 0.001), suggesting that more modern cultivars tend to present higher yields. The correlation between year of release and grain yield was stronger when considering all 61 cultivars than when looking solely at cultivars released post‐1980 (*n* = 56), post‐1990 (*n* = 45), or post‐2000 (*n* = 36; Figure 3). The slope of the regression reflects the relative improvement of approximately 0.5 t ha^−1^ decade^−1^, with the slight increase in slope in more recent releases suggesting somewhat faster rates of grain yield increase.

Conversely, year of release was not significantly correlated to yield stability, as indicated by the yield variation over the three seasons (*p* > 0.05), suggesting that in the past decades wheat breeding has been successful in improving yield potential, but not necessarily yield stability. In this analysis, stability is considered as a general trait related to yield variation. An alternative approach is to consider crop performance under suboptimal conditions, such as those experienced in 2013 and 2014. The suboptimal performance index, estimated based on the average relative grain yield for 2013 and 2014, was positively correlated to year of release (*r *=* *0.50, *p* < 0.001), indicating that modern wheat cultivars tend to perform consistently better, despite the environmental conditions they are exposed to.

### 3.3 Genetic similarity partially explained grain yield patterns

**Figure 4:**
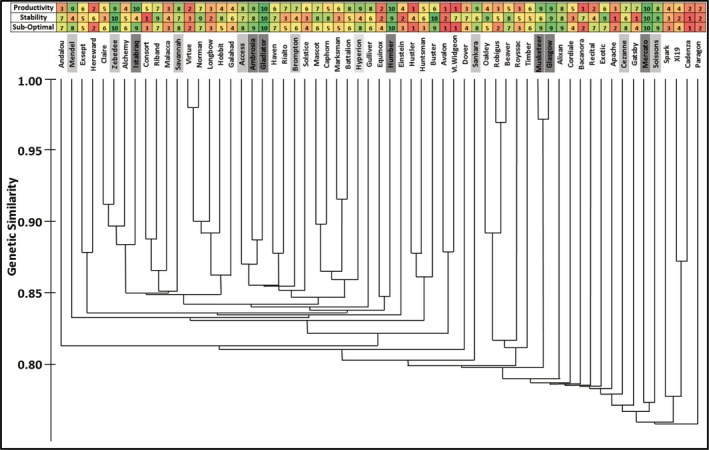
Crop performance indices and genetic similarities based on marker analysis for 61 UK field‐grown wheat cultivars over three consecutive seasons (2012, 2013, and 2014). Productivity, rank for average relative grain yield for the three seasons; stability, rank for the ratio between the cultivar grain yield standard deviation and the average grain yield for the 61 cultivars over the three seasons; suboptimal performance, rank for average relative grain yield for 2013 and 2014. Ranks vary from 1 (dark red) to 10 (dark green). Cultivars in light gray presented relative grain yield always at or above the 61‐cultivar average; cultivars in dark gray presented relative grain yields always at or above the 61‐cultivar average and were in the upper quartile for 2013 and 2014

Crop performance in terms of grain yield pattern over the three seasons was correlated to the marker‐based genetic similarity (Figure 4). The genetic similarity was estimated with reference to the cultivar Gladiator, due to its superior average relative grain yield over the three seasons, and was positively correlated to the productivity (*r *=* *0.35, *p* < 0.01) and the suboptimal performance indices (*r *=* *0.37, *p* < 0.01). No significant correlation was observed between genetic similarity and yield stability. The link between the genetic similarity and yield performance can also be observed by the proximity of some cultivars in the cluster, such as Ambrosia and Gladiator or Musketeer and Glasgow (Figure 4).

## 4 DISCUSSION

Assessment of crop productivity, yield stability, and relative performance under suboptimal conditions enabled the identification of four high‐performing wheat cultivars. To enable this analysis, 61 wheat cultivars of the ERYCC panel were grown at the Rothamsted farm, in the UK, for three consecutive seasons: 2012, 2013, and 2014. Genetic variation was observed in grain yield, yield stability, and performance under suboptimal conditions. Genetic similarities partially explained yield performance.

Understanding yield performance of wheat cultivars under different environmental conditions is crucial to select potential targets for breeding programs and to predict plant behavior in future climatic conditions. According to Semenov, Stratonovitch, Alghabari, and Gooding (2014), modeling of future climatic conditions suggests that the wheat cycle in Europe will tend to be shorter with later sowing and earlier harvest. Later sowing of winter wheat in temperate climates may be necessary so that the crop experiences ideal temperatures (Waha, van Bussel, Müller, & Bondeau, 2012). Warm temperatures early in the cycle will otherwise result in the formation of dense canopies before winter commences and lead to frost damage (AHDB, 2011). In addition, drought and heat stress are more likely to occur at the late stages of the crop cycle. These conditions will impact on the duration of critical developmental stages, such as grain development, limiting yields. The above‐cited conditions are particularly similar to the ones observed in the present study in the 2013 season, and less so in the 2014 season. The negative impact of late sowing in grain yield observed in the present study was also reported by Ghaffari, Cook, and Lee (2002). Finding the best sowing date to maintain yield stability in the changing climate may prove challenging.

The combination of high yield and yield stability is a desired trait for crop breeding. The hypothesis that yield stability in suboptimal conditions is linked to yield penalties in optimal conditions was not supported by the results reported herein. There was a positive correlation between productivity and stability (*r *=* *0.40, *p* < 0.001; Figure 2), and the performance of the highlighted cultivars, Gladiator, Humber, Mercato, and Zebedee (Figure 2), suggested combined high and stable yields in these cultivars in the three seasons and respective environmental conditions. Future investigation of yield stability in conditions far more stressful than those observed in the present study may yield different conclusions.

Gladiator, Humber, Mercato, and Zebedee attained grain yields always at or above the 61‐cultivar average and presented relatively low variation in yield over the seasons. For instance, Gladiator yielded ca. 15% more than the 61‐cultivar average and presented a yield variation of ca. 13% over the three years. Zebedee was more stable, but yielded less than Gladiator on average. Istabraq had an outstanding performance in terms of yield, but was relatively less stable with ca. 25% variation. On the other hand, Buster was the most stable cultivar (8% variation), but yielded 16% less than the 61‐cultivar average in 2012 (Figure 2 and Table 1), supporting the contention that some, but not all, cultivars with stable yields are penalized in good years, such as 2012.

The four best cultivars in terms of grain yield and stability were all released on or after 2000 (Gladiator, 2005; Humber, 2006; Mercato, 2005; Zebedee, 2000), but there was no correlation between year of release and stability for the whole population. The presence of cultivars released before the Green Revolution could have biased the analysis, but the lack of correlation was consistent when considering only cultivars released more recently.

The cultivars Glasgow, Istabraq, Mercato, Musketeer, Ambrosia, Gladiator, and Humber (Figure 2) were the only ones in the upper quartile for grain yield in 2013 and 2014 (Table 1), showing greater resilience to the conditions faced in the two seasons. However, Ambrosia did not attain as high yield as the others, Istabraq was less stable, and Musketeer was intermediate between the two. This highlights the importance of choosing cultivars not just for one characteristic, but a combination of high yield, performance under adverse environmental conditions and yield stability, as previously suggested by Powell, Ji, Ravash, Edlington, and Dolferus (2012). Broad‐sense heritability of grain yield helped to understand the impact of the environmental conditions on yield variation over the three seasons. Carmo‐Silva et al. (2017) reported broad‐sense heritability of 0.89 and 0.58 for grain yield in 2012 and 2013, respectively. Those values were different from the ones reported in this study (Table 2), although, in both studies, they suggest greater genetic control of grain yields in 2012, compared to 2013. The suboptimal conditions observed in 2013 impacted on the final grain yield and increased the yield gap. Grain yield is a complex trait driven by a combination of multiple genes. Evaluating the heritability of less complex traits related to yield stability could help understand plant performance in different environmental conditions. The differences in the reported heritability values herein and in Carmo‐Silva et al. (2017) are likely to be related to the subjectivity of the decision on the best model on the REML (reduced maximum likelihood) method to account for spatial variation on the field data. Different analyzers might end up with different models, impacting on the heritability calculations.

Revealing the genetic and physiological background of yield potential, stability, and performance under suboptimal conditions could enhance the understanding of the different strategies adopted by cultivars to reach a better performance (Reynolds & Langridge, 2016). In the specific case of the duration of grain development, which is critical for final grain yield (Evans & Fischer, 1999), cultivars with superior performance might be more efficient in their use of resources for grain filling (Hunt, van der Poorten, & Pararajasingham, 1990) or able to extend the duration of this growth stage despite sowing date or accumulated rainfall patterns (Richter & Semenov, 2005).

Carmo‐Silva et al. (2017) reported that, for the 2013 season, Gladiator was the highest yielding cultivar and presented the highest flag leaf longevity (from Zadoks 4 to Senescence index 5) across the ERYCC panel. This cultivar also showed no decrease in flag leaf photosynthetic rate from pre‐ to postanthesis, presenting one of the highest flag leaf photosynthetic rates at postanthesis. These results suggest a sustained supply of photoassimilates from the flag leaf to the grain, despite the suboptimal conditions in 2013. Pennacchi et al. (2018) reported the positive correlation between flag leaf photosynthetic levels and stay green to wheat yield, and Lopes and Reynolds (2012) also reported a correlation between flag leaf duration and grain yield in wheat under drought and/or heat stress. In addition to that, Carmo‐Silva et al. (2017) also showed that Mercato and Zebedee had high flag leaf photosynthetic rate at preanthesis, which could have promoted stem reserve accumulation. During the grain development stage, these reserves could have been reallocated to the grain, explaining their higher yields in 2013. The importance of stem reserve remobilization to the grain under heat stress in wheat was reported by Blum, Sinmena, Mayer, Golan, and Shpiler (1994) and Tahir and Nakata (2005). Although the temperatures were not as extreme as in those studies (over 38°C), in 2013 a combined effect of high temperature and radiation with low water availability at grain development could have increased reserve reallocation compared to 2012 (Table 3). Alternative strategies such as those observed for Gladiator and for Mercato/Zebedee could improve the grain filling rate under suboptimal conditions and impact on grain yield and yield stability.

**Table 3:** Environmental conditions at grain development stage for 61 UK field‐grown wheat cultivars over three seasons (2012, 2013, and 2014)

	2012	2013	2014
Duration of grain development (days)	47	31	37
Average maximum temperature (°C)	20.0	25.5	21.7
Highest maximum temperature (°C)	28.0	31.4	30.1
Daily accumulated degrees day (°C day/day)	16.1	19.2	16.8
Daily accumulated rainfall (mm/day)	3.6	1.6	1.1
Daily accumulated radiation (MJ m^−2^ day^−1^)	16.6	21.3	18.7

In addition to flag leaf photosynthesis, ear photosynthesis also contributes to grain filling (Sanchez‐Bragado, Molero, Reynolds, & Araus, 2014; Zhou et al., 2016), especially under abiotic stress (Abbad, El Jaafari, Bort, & Araus, 2004). Moreover, the source–sink balance can regulate photosynthesis (Paul & Foyer, 2001) and grain filling (Paul, Oszvald, Jesus, Rajulu, & Griffiths, 2017), impacting directly on wheat yields (Valluru, Reynolds, & Lafarge, 2015). Finally, it is noteworthy that to ensure food security both grain quantity and quality need to be considered (Shewry, 2007).

Although more modern cultivars presented higher yields in this study and the rate of yield increase in the ERYCC panel has been sustained around 0.5 t ha^−1^ decade^−1^ (as reported in this study and by Clarke et al., 2012), the lack of yield stability and the negative impact of suboptimal conditions on grain yield production for most wheat cultivars may contribute to explain the recent plateauing of wheat yields at around 8 t/ha, at the farm level, in the UK since 1996 (Knight et al., 2012).

## 5 CONCLUSION

This study identified four UK modern cultivars, Gladiator, Humber, Mercato, and Zebedee, with relatively high grain yield potential combined with stable yields across three seasons characterized by contrasting environmental conditions for the UK. Further study of the genetic and physiological basis of combined yield potential and stability using these cultivars is warranted. The findings are relevant to the development of mapping populations in breeding programs aimed at increasing yield potential and climate resilience for temperate regions in order to achieve sustained increases in yields at the farm level.

## References

[fes3147-bib-0001] Abbad, H. , El Jaafari, S. , Bort, J. P. , & Araus, J. L. (2004). Comparison of flag leaf and ear photosynthesis with biomass and grain yield of durum wheat under various water conditions and genotypes. Agronomie, 24, 19–28. 10.1051/agro:2003056

[fes3147-bib-0002] AHDB (2011). Wheat growth guide. Warwickshire, UK: Agriculture and Horticulture Development Board.

[fes3147-bib-0003] Araus, J. L. , Slafer, G. A. , Royo, C. , & Serret, M. D. (2008). Breeding for yield potential and stress adaptation in cereals. Critical Reviews in Plant Science, 27, 377–412. 10.1080/07352680802467736

[fes3147-bib-0004] Avery, B. W. , & Catt, J. A. (1995). The soil at Rothamsted. Map prepared by E M Thompson and the Soil Survey and Land Research Centre, Cranfield University, Lawes Agricultural Trust, Harpenden, UK

[fes3147-bib-0005] Berry, E. M. , Dernini, S. , Burlingame, B. , Meybeck, A. , & Conforti, P. (2015). Food security and sustainability: Can one exist without the other? Public Health Nutrition, 18, 2293–2302. 10.1017/S136898001500021X 25684016 PMC10271846

[fes3147-bib-0006] Blum, A. , Sinmena, B. , Mayer, J. , Golan, G. , & Shpiler, L. (1994). Stem reserve mobilisation supports wheat grain filling under heat stress. Australian Journal of Plant Physiology, 21, 771–781. 10.1071/PP9940771

[fes3147-bib-0007] Braun, H. J. , Atlin, G. , & Payne, T. (2010). Multi‐location testing as a tool to identify plant response to global climate change In Reynolds M. P. (Ed.), Climate change and crop production (pp. 115–138). Oxfordshire, UK: CABI.

[fes3147-bib-0008] Carmo‐Silva, E. , Andralojc, P. J. , Scales, J. C. , Driever, S. M. , Mead, A. , Lawson, T. , … Parry, M. A. J. (2017). Phenotyping of field‐grown wheat in the UK highlights contribution of light response of photosynthesis and flag leaf longevity to grain yield. Journal of Experimental Botany, 68, 3473–3486. 10.1093/jxb/erx169 28859373 PMC5853948

[fes3147-bib-0009] Ciais, P. , Reichstein, M. , Viovy, N. , Granier, A. , Ogee, J. , Allard, V. , … Valentini, R. (2005). Europe‐wide reduction in primary productivity caused by the heat and drought in 2003. Nature, 437, 529–533. 10.1038/nature03972 16177786

[fes3147-bib-0010] Clarke, S. , Sylvester‐Bradley, R. , Foulkes, J. , Ginsburg, D. , Gaju, O. , Werner, P. , … Smith‐Reeve, L. (2012). Adapting wheat to global warming or ‘ERYCC’ – Earliness and Resilience for Yield in a Changing Climate. HGCA Project Report No. 496

[fes3147-bib-0011] Cullis, B. R. , Smith, A. B. , & Coombes, N. E. (2006). On the design of early generation variety trials with correlated data. Journal of Agricultural, Biological, and Environmental Statistics, 11, 381–393. 10.1198/108571106X154443

[fes3147-bib-0012] Driever, S. M. , Lawson, T. , Andralojc, P. J. , Raines, C. A. , & Parry, M. A. J. (2014). Natural variation in photosynthetic capacity, growth, and yield in 64 field‐grown wheat genotypes. Journal of Experimental Botany, 65, 4959–4973. 10.1093/jxb/eru253 24963002 PMC4144772

[fes3147-bib-0013] Evans, L. T. , & Fischer, R. A. (1999). Yield potential: Its definition, measurement, and significance. Crop Science, 39, 1544–1551. 10.2135/cropsci1999.3961544x

[fes3147-bib-0014] Fischer, R. A. , Byerlee, D. , & Edmeades, G. O. (2014). Crop yields and global food security: will yield increase continue to feed the world? ACIAR Monograph No. 158. Canberra

[fes3147-bib-0015] Fischer, R. A. , & Edmeades, G. O. (2010). Breeding and cereal yield progress. Crop Science, 50, 85–98. 10.2135/cropsci2009.10.0564

[fes3147-bib-0016] Gao, H. , Yang, R. , Zhao, W. , & Pan, Y. (2005). A new method for calculating molecular genetic similarity. Nature and Science, 3, 71–74.

[fes3147-bib-0017] Ghaffari, A. , Cook, H. F. , & Lee, H. C. (2002). Climate change and winter wheat management: A modelling scenario for South‐Eastern England. Climatic Change, 55(509), 533.

[fes3147-bib-0018] Godfray, H. C. J. , Beddington, J. R. , Crute, I. R. , Haddad, L. , Lawrence, D. , Muir, J. F. , … Toulmin, C. (2010). Food Security: The challenge of feeding 9 billion people. Science, 327, 812–818. 10.1126/science.1185383 20110467

[fes3147-bib-0019] Gregory, P. J. , & George, T. S. (2011). Feeding nine billion: The challenge to sustainable crop production. Journal of Experimental Botany, 62, 5233–5239. 10.1093/jxb/err232 21841178

[fes3147-bib-0021] Hunt, L. A. , van der Poorten, G. , & Pararajasingham, S. (1990). Postanthesis temperature effects on duration and rate of grain filling in some winter and spring wheats. Canadian Journal of Plant Sciences, 71, 609–617.

[fes3147-bib-0022] Knight, S. , Kightley, S. , Bingham, I. , Hoad, S. , Lang, B. , Philpott, H. , … Ball, B. (2012). Desk study to evaluate contributory causes of the current ‘yield plateau’ in wheat and oilseed rape. HGCA Project Report No. 502

[fes3147-bib-0023] Lobell, D. B. , Cassman, K. G. , & Field, C. B. (2009). Crop yield gaps: Their importance, magnitudes, and causes. Annual Review of Environment and Resources, 34, 179–204. 10.1146/annurev.environ.041008.093740

[fes3147-bib-0024] Lopes, M. S. , & Reynolds, M. P. (2012). Stay‐green in spring wheat can be determined by spectral reflectance measurements (normalized difference vegetation index) independently from phenology. Journal of Experimental Botany, 63, 3789–3798. 10.1093/jxb/ers071 22412185 PMC3388823

[fes3147-bib-0025] McMaster, G. S. , & Smika, D. E. (1988). Estimation and evaluation of winter wheat phenology in the central great plains. Agricultural and Forestry Meteorology, 43, 1–18. 10.1016/0168-1923(88)90002-0

[fes3147-bib-0026] Ober, E. S. , Werner, P. , Flatman, E. , Angus, B. , Jack, P. , & Tapsell, C. (2013). Improving water use efficiency and drought tolerance in UK winter wheats. HGCA Project Report No. 476

[fes3147-bib-0027] Paul, M. J. , & Foyer, C. H. (2001). Sink regulation of photosynthesis. Journal of Experimental Botany, 52, 1383–1400. 10.1093/jexbot/52.360.1383 11457898

[fes3147-bib-0028] Paul, M. J. , Oszvald, M. , Jesus, C. , Rajulu, C. , & Griffiths, C. A. (2017). Increasing crop yield and resilience with trehalose 6‐phosphate: Targeting a feast–famine mechanism in cereals for better source–sink optimization. Journal of Experimental Botany, 68, 4455–4462. 10.1093/jxb/erx083 28981769

[fes3147-bib-0029] Pennacchi, J. P. , Carmo‐Silva, E. , Andralojc, P. J. , Feuerhelm, D. , Powers, S. J. , & Parry, M. A. J. (2018). Dissecting wheat grain yield drivers in a mapping population in the UK. Agronomy, 8, 94–108. 10.3390/agronomy8060094

[fes3147-bib-0030] Pingali, P. L. (2006). Westernization of Asian diets and the transformation of food systems: Implications for research and policy. Food Policy, 32, 281–298.

[fes3147-bib-0031] Powell, N. , Ji, X. , Ravash, R. , Edlington, J. , & Dolferus, R. (2012). Yield stability for cereals in a changing climate. Functional Plant Biology, 39, 539–552. 10.1071/FP12078 32480806

[fes3147-bib-0032] Ray, D. K. , Gerber, J. S. , MacDonald, G. K. , & West, P. C. (2015). Climate variation explains a third of global crop yield variability. Nature Communications, 6, 1–9. 10.1038/ncomms6989 PMC4354156 25609225

[fes3147-bib-0033] Reynolds, M. , & Langridge, P. (2016). Physiological breeding. Current Opinion in Plant Biology, 31, 162–171. 10.1016/j.pbi.2016.04.005 27161822

[fes3147-bib-0034] Richter, G. M. , & Semenov, M. A. (2005). Modelling impacts of climate change on wheat yields in England and Wales: Assessing drought risks. Agricultural Systems, 84, 77–97. 10.1016/j.agsy.2004.06.011

[fes3147-bib-0035] Robertson, M. , Kirkegaard, J. , Rebetzke, G. , Llewellyn, R. , & Wark, T. (2016). Prospects for yield improvement in the Australian wheat industry: A perspective. Food and Energy Security, 5, 107–122. 10.1002/fes3.81

[fes3147-bib-0036] Romagosa, I. , & Fox, P. N. (1993). Genotype‐environment interaction and adaption In Hayward M. D., Bosemark N. O., & Romagosa I. (Eds.), Plant breeding: Principles and perspectives (pp. 373–390). Heidelberg, Germany: Chapman and Hall.

[fes3147-bib-0037] Sanchez‐Bragado, R. , Molero, G. , Reynolds, M. P. , & Araus, J. L. (2014). Relative contribution of shoot and ear photosynthesis to grain filling in wheat under good agronomical conditions assessed by differential organ δ^13^C. Journal of Experimental Botany, 65, 5401–5413. 10.1093/jxb/eru298 25053645 PMC4157716

[fes3147-bib-0038] Schmidhuber, J. , & Tubiello, F. N. (2007). Global food security under climate change. Proceedings of the National Academy of Sciences of the United States of America, 104, 19703–19708. 10.1073/pnas.0701976104 18077404 PMC2148361

[fes3147-bib-0039] Semenov, M. A. , Stratonovitch, P. , Alghabari, F. , & Gooding, M. J. (2014). Adapting wheat in Europe for climate change. Journal of Cereal Science, 59, 245–256. 10.1016/j.jcs.2014.01.006 24882934 PMC4026126

[fes3147-bib-0040] Shewry, P. R. (2007). Improving the protein content and composition of cereal grain. Journal of Cereal Science, 46, 239–250. 10.1016/j.jcs.2007.06.006

[fes3147-bib-0041] Tahir, I. S. A. , & Nakata, N. (2005). Remobilization of nitrogen and carbohydrate from stems of bread wheat in response to heat stress during grain filling. Journal of Agronomy and Crop Science, 191, 106–115. 10.1111/j.1439-037X.2004.00127.x

[fes3147-bib-0042] Tester, M. , & Langridge, P. (2010). Breeding technologies to increase crop production in a changing world. Science, 327, 818–822. 10.1126/science.1183700 20150489

[fes3147-bib-0043] Tilman, D. , & Clark, M. (2015). Food, agriculture & the environment: Can we feed the world & save the earth? American Academy of Arts & Sciences, 144, 1–23.

[fes3147-bib-0044] Valluru, R. , Reynolds, M. P. , & Lafarge, T. (2015). Food security through translational biology between wheat and rice. Food and Energy Security, 4, 203–218. 10.1002/fes3.71

[fes3147-bib-0045] Waha, K. , van Bussel, L. G. J. , Müller, C. , & Bondeau, A. (2012). Climate‐driven simulation of global crop sowing dates. Global Ecology and Biogeography, 21, 247–259. 10.1111/j.1466-8238.2011.00678.x

[fes3147-bib-0046] Zadoks, I. C. , Chang, T. T. , & Konzak, C. F. (1974). A decimal code for the growth stages of cereals. Weed Research, 14, 415–421. 10.1111/j.1365-3180.1974.tb01084.x

[fes3147-bib-0047] Zhou, B. , Serret, M. B. , Elazab, A. , Pie, J. B. , Araus, J. L. , Aranjuelo, I. , & Sanz‐Sáez, A. (2016). Wheat ear carbon assimilation and nitrogen remobilization contribute significantly to grain yield. Journal of Integrative Plant Biology, 58, 914–926. 10.1111/jipb.12478 26990448

