# Supplementary material for: Stability of wheat grain yields over three field seasons in the UK
Source: Food Energy Secur. 2018 Sep 19;8(2):e00147. doi: 10.1002/fes3.147 (PMC6582621; doi:10.1002/fes3.147)
Supplement: Supplementary file 1 [file FES3-8-na-s001.docx]

**Table S1.** The 61 cultivars of the ERYCC panel grown at Rothamsted Research from 2012 to 2014 and respective, year of release, origin, habit, indication of UK wheat market (when applicable), grouping and parentage.

| ERYCC Cultivar | Release^a^ | Origin^a^ | Habit^a^ | UK Market | ERYCC  Group | Parentage^a^ |
| --- | --- | --- | --- | --- | --- | --- |
| Access | 2002 | UK | Winter |  | Modern | 90-15 x 91-6 |
| Alchemy | 2002 | UK | Winter | Feed | Modern | CLAIRE x (CONSORT x WOODSTOCK) |
| Alixan | 2005 | France | Winter |  | Phenology | n/a |
| Ambrosia | 2005 | UK | Winter |  | Modern | (CANTATA(SIB) x GENESIS) x PINDER |
| Andalou | 2002 | France | Winter |  | Phenology | n/a |
| Apache | 1998 | France | Winter |  | Phenology | AXIAL x NRPB-84-4233 |
| Avalon | 1980 | UK | Winter | Bread | Historical | MARIS-PLOUGHMAN x BILBO; TJB-30-148 x BILBO |
| Bacanora | 1988 | Mexico | Spring |  | Phenology | (JUPATECO-73 x (SIB)BLUEJAY) x URES-81 |
| Battalion | 2006 | UK | Winter |  | Modern | 98-ST-08 x AARDVARK |
| Beaver | 1989 | UK | Winter | Feed | Historical | (HEDGEHOG x NORMAN) x MOULIN |
| Brompton | 2005 | UK | Winter |  | Modern | CWW-92-1 x CAXTON |
| Buster | 1992 | UK | Winter |  | Phenology | BRIMSTONE x PARADE |
| Cadenza | 1992 | France | Spring |  | Phenology | TONIC x AXONA; TONIC x AXONA |
| Caphorn | 2000 | UK | Winter |  | Phenology | (S-14579-454 x RIALTO) x BEAUFORT |
| Cezanne | 1997 | France | Winter |  | Phenology | THESEE x 87-B-29 |
| Claire | 1999 | UK | Winter | Feed | Modern | WASP x FLAME |
| Consort | 1993 | UK | Winter | Feed | Modern | (RIBAND(SIB) x FRESCO) x RIBAND |
| Cordiale | 2007 | UK | Winter | Bread | Phenology | (REAPER x CADENZA) x MALACCA |
| Dover | 2005 | UK | Winter |  | Modern | (BISCAY x AARDVARK) x F-86-Z-46-6-2 |
| Einstein | 2007 | UK | Winter | Bread | Modern | NSL-WW-91-1670 x NSL-WW-90-1282 |
| Equinox | 1995 | UK | Winter |  | Historical | CWW-4442-4 x (RENDEZVOUS x OBELISK); CWW-4442-64 x (RENDEZVOUS x OBELISK) |
| Exotic | 2005 | France | Winter |  | Phenology | n/a |
| Exsept | 2001 | Germany | Winter |  | Phenology | HEREWARD x TORCH |
| Galahad | 1983 | UK | Winter | Feed | Historical | (JOSS-CAMBIER x DURIN) x (SIB)HOBBIT |
| Gatsby | 2007 | UK | Winter |  | Phenology | NELSON x WASMO |
| Gladiator | 2005 | UK | Winter |  | Modern | FALSTAFF x SHANNON |
| Glasgow | 2006 | France | Winter |  | Modern | (RITMO x ZE.90-2666) x ZE-11658 |
| Gulliver | 2006 | UK | Winter |  | Modern | SHAMROCK x AARDVARK |
| Haven | 1988 | UK | Winter |  | Historical | (HEDGEHOG x NORMAN) x MOULIN; MANDATE x MOULIN |
| Hereward | 1989 | UK | Winter | Bread | Modern | NORMAN x DISPONENT; DISPONENT x NORMAN; NORMAN(SIB) x DISPONENT |
| Hobbit | 1977 | UK | Winter |  | Historical | [PROFESSEUR MARCHAL x (MARNE-DESPREZ x VG-9144)] x TJB 16 |
| Humber | 2006 | UK | Winter |  | Modern | ANGLO x KRAKATOA |
| Hustler | 1978 | UK | Winter |  | Historical | MARIS-HUNTSMAN x MARIS-DURIN |
| Hyperion | 2012 | France | Winter |  | Phenology | AARDVARK x (CONSORT x WOODSTOCK) |
| Istabraq | 2004 | UK | Winter |  | Modern | CONSORT x CLAIRE |
| Longbow | 1980 | UK | Winter |  | Historical | TJB-268-175 x HOBBIT |
| Malacca | 1997 | UK | Winter | Bread | Modern | (RIBAND x RENDEZVOUS) x APOSTLE; RIBAND x (RENDEZVOUS x APOSTLE) |
| Maris Huntsman | 1972 | UK | Winter | Feed | Historical | [(C1.12633 x CAPPELLE-DESPREZ) x HYBRID-46] x PROFESSEUR-MARCHAL |
| Maris Widgeon | 1964 | UK | Winter | Bread | Historical | HOLDFAST x CAPPELLE-DESPREZ |
| Marksman | 2007 | UK | Winter |  | Modern | 98-ST-08 x AARDVARK |
| Mascot | 2005 | UK | Winter |  | Modern | REAPER x RIALTO |
| Mendel | 2005 | Sweden | Winter |  | Phenology | RED-STANDARD x TRIFOLIUM-14; TRIFOLIUM x WEIBULLS-IDUNA; STANDARD x TRIFOLIUM-14 |
| Mercato | 2005 | France | Winter |  | Phenology | n/a |
| Musketeer | 2007 | UK | Winter |  | Modern | (WICKHAM(SIB) x CHARGER) x AARDVARK |
| Norman | 1981 | UK | Winter | Feed | Historical | TJB-268-175 x (SIB)HOBBIT |
| Oakley | 2008 | UK | Winter | Feed | Modern | (AARDVARK x ROBIGUS) x ACCESS |
| Paragon | 1998 | UK | Spring |  | Phenology | CSW-1724-19-5-68 x (AXONA x TONIC) |
| Recital | 1986 | France | Winter |  | Phenology | MEXIQUE-267(R-267) x 9369; 9369 x R-267 |
| Rialto | 1993 | UK | Winter | Bread | Historical | HAVEN(SIB) x (SIB)FRESCO |
| Riband | 1987 | UK | Winter | Feed | Historical | NORMAN x TW-275; NORMAN x (MARIS-HUNTSMAN x TW-161) |
| Robigus | 2005 | UK | Winter | Feed | Modern | Z-836 x 1366; Z-836 x PUTCH |
| Royssac | 2003 | France | Winter |  | Phenology | n/a |
| Sankara | 2004 | France | Winter |  | Phenology | n/a |
| Savannah | 1998 | UK | Winter |  | Historical | RIBAND x BRIGADIER |
| Soissons | 1987 | France | Winter |  | Phenology | IENA(JENA) x (HYBRIDE-NATUREL)HN-35 |
| Solstice | 2002 | Netherlands | Winter | Bread | Modern | VIVANT x RIALTO |
| Spark | 1991 | UK | Spring |  | Phenology | MOULIN x TONIC |
| Timber | 2006 | France | Winter |  | Phenology | TERRIER x HAMAC |
| Virtue | 1979 | UK | Winter |  | Historical | MARIS-HUNTSMAN x MARIS-DURIN |
| Xi19 | 2002 | UK | Winter |  | Modern | (CADENZA x RIALTO) x CADENZA |
| Zebedee | 2000 | UK | Winter |  | Modern | CLAIRE x NELSON |

^a^ Data from: Genetic Resources Information System for Wheat and Triticale (CIMMYT): <http://wheatpedigree.net/>; and The Scottish Wheat Variety Database: <http://wheat.agricrops.org/varietyindex.php?page_no=1>

**Table S2.** Rothamsted farm practices details for the three seasons.

| Date | Application | Rate/Units |
| --- | --- | --- |
| 2012 | | |
| 30/08/2011 | Sprayed Statis 360 | 1.5 lt/ha |
| 14/10/2011 | Sprayed Liberator | 0.6 lt/ha |
| 28/11/2011 | Sprayed Hallmark with Zeon Technology | @50 ml/ha |
| 13/03/2012 | Fert spread, Doubletop | @185 kg/ha |
| 14/04/2012 | Sprayed Cherokee and Justice | Ch@1.0 l/ha, Ju@0.125 l/ha |
| 04/05/2012 | Sprayed Agriguard Chlormequat 720, Bravo 500, Tracker, Ally Max and Starane2 | Ag@2.25 l/ha, Br@1.0 l/ha, Tr@1.0 l/ha, Al@42 g/ha, St@0.5 l/ha |
| 21/05/2012 | Applied Nitram | @174 kgs/ha |
| 24/05/2012 | Sprayed w/ Comet, Bravo, Ignite/Opus | Co@0.6 l/ha, Br@1.0 l/ha, Ig@ 1.0 l/ha, Op@0.8 l/ha |
| 13/06/2012 | Sprayed w/ Cello | @0.55 l/ha |
| 2013 | | |
| 07/03/2013 | Applied Slug Pellets | @5 kg/ha |
| 16/04/2013 | Applied DoubleTop Fertilizer | @222 kg/ha |
| 30/04/2013 | Applied Nitram | @348 kg/ha |
| 16/05/2013 | Sprayed AllyMax, Kingdom, Bravo500, NewCycocel, HatchetExtra | AM@42 g/ha, Ki@1.25 l/ha, Br500@1.0l/ha, NCy@2.0 l, Hatc@1.0 l/ha |
| 06/06/2013 | Sprayed Ignite, Comet, Topik, Zarado | Ign@1.2 l/ha, Com@0.4 l/ha, Top@0.15 l/ha, Zar@1.0 l/ha |
| 14/06/2013 | Sprayed Cyflamid | @200 ml/ha |
| 19/06/2013 | Sprayed Cello, Corbel | Cel@0.55 l/ha, Cor@0.5 l/ha |
| 2014 | | |
| 27/11/2013 | Applied Major Slug Pellets | @4 kg/ha |
| 03/12/2013 | Sprayed Samurai, Liberator, Stomp | Sam@1.5 l/ha, Lib@0.6 l/ha, Sto@1.7 l/ha |
| 02/04/2014 | Sprayed Artemis, Bravo 500, Moddus and BASF 3C 720 | Art@1 l/ha, Bra@1 l/ha, Mod@150 ml/ha, BASF@1.25 l/ha |
| 25/04/2014 | Applied Nitram | @174 kg/ha |
| 16/05/2014 | Sprayed Vortex | @1.5 lt/ha |
| 06/06/2014 | Sprayed Cello | @550 ml/ha |
